# Small airway dysfunction in patients with cough variant asthma: a retrospective cohort study

**DOI:** 10.1186/s12890-021-01419-4

**Published:** 2021-02-03

**Authors:** Jie Gao, Haigui Wu, Feng Wu

**Affiliations:** Department of Pulmonary and Critical Care Medicine, Huizhou the Third People’s Hospital, Guangzhou Medical College, 1# Xuebei Ave., Huizhou, 516002 Guangdong China

**Keywords:** Sputum cells, Bronchial hyper-responsiveness, Cough variant asthma, Classic asthma

## Abstract

**Background:**

Cough variant asthma (CVA) is one of the special populations of asthma. The aim of the study was to compare small airways, the degree of bronchial hyperresponsiveness (BHR) and airway inflammatory subtypes between CVA and classic asthma (CA), and investigate the relationship between these markers to determine the accuracy as indicators of CVA.

**Methods:**

A total of 825 asthmatic patients participated in the study and 614 were included. 614 patients underwent spirometry and a bronchial challenge with methacholine and 459 patients performed induction sputum cell test.

**Results:**

The number of CVA patients showed less small airway dysfunction than those of CA patients (*p* < 0.005). The degree of small airways dysfunction was higher in the CA group compared with the CVA group (*p* < 0.001). Small airways dysfunction was severer in the eosinophilic airway inflammatory subtype compared with other subtypes (*p* < 0.05).The area under curve of MMEF, FEF_50_ and FEF_75_ (% predicted) was 0.615, 0.621, 0.606, respectively. 0.17mcg of PD_20_ and 4.7% of sputum eosinophils was the best diagnostic value for CVA with an AUC of 0.582 and 0.575 (*p* = 0.001 and *p* = 0.005, respectively).

**Conclusions:**

The eosinophilic airway inflammatory subtype may be increased small airway dysfunction. The value of small airways, BHR and induction sputum cells in CVA prediction, which reflected significant, but not enough to be clinically useful.

## Background

Asthma is a heterogeneous disorder disease with chronic airway inflammation of bronchial hyperresponsiveness (BHR) to a variety of stimuli, and variable expiratory airflow limitation that is often reversible either spontaneously or as a result of therapy [1, 2]. “Classic asthma (CA)” is based on the characteristics and the respiratory symptoms, such as shortness of breath tightness, wheeze and cough [1].

The original definition of cough variant asthma (CVA) was described by Glauser and later by Carrao, McFadden in 1972 and 1975, 1979 [3–5]. They described cough as asthmatic patient sole presenting symptom, and the symptom improved with bronchodilators alone. The European and American guidelines do not discuss specific diagnostic criteria of CVA, but they recommend that the CVA diagnosis should be determined according to the value of BHR and the therapeutic response [1]. The Chinese Cough guidelines (2016) take the detailed diagnostic criteria and treatment of CVA, which is the BHR and successful treatment of bronchodilators and/or inhaled corticosteroids as the basic diagnostic criteria [6].

Small airways were defined as the bronchial less than 2 mm in internal diameter [7]. They played a role in the pathobiology of asthma and have a distinct role in specific disease phenotypes, although they are involved in half of all cases of asthma [8–10]. The severity of asthma was also associated with inflammatory changes and functional alterations in the small airways [11, 12]. Sputum induction (inhalation hypertonic saline) is a noninvasive technique that has been valid for studying inflammatory cells in airways. In particular, sequential inductions performed after short intervals of time (20–30 min), may provide useful information on distal airway inflammation [13, 14]. The role of the small airways in asthma is increasingly recognized as a potential target in optimal control of the disease. Therefore, this study aims to explore the validity of small airways, the degree of BHR and airway inflammatory subtypes in the diagnosis of CVA.

## Methods

### Study design

This retrospective study was conducted in the Huizhou Third People’s Hospital of Guangzhou Medical University between January 2018 and April 2019 in China. The primary diagnosis included history inquiring and physical examination, spirometry, a bronchial challenge with methacholine, sputum induction and CT of the chest. The final diagnosis was made based on clinical manifestation, examination findings and a positive response to therapy. No prospective data was collected and all data was obtained retrospectively (from the hospital medical records).

CA patients were diagnosed according to (1) a clinical record of recurrent dyspnea or/and cough episodes, wheezing, chest tightness; (2) variable airflow limitation employed by BHR test or bronchodilator reversibility test; (3) a positive therapeutic response; (4) diagnosis of CA requires all the three points, which satisfied those of the Chinese National Guidelines on Diagnosis and Management of Asthma (2016) [15].

CVA patients were described (1) chronic cough (lasting more than 8 weeks without specific cause) as the only symptom; (2) the cough was mainly nocturnal and usually dry or productive with minimal amounts of clear sputum; (3) Normal baseline lung function, some might include small airways dysfunction; (4) a positive test for (direct) BHR; (5) No other cause of chronic cough; (6) a positive therapeutic response [6].

Inclusion criteria were: age greater than 14 years old; diagnosis of CA and CVA according to the guidelines criteria of China [6, 15]; uncontrolled in the stage; no other apparent causes of cough; not used any corticosteroid (ICS) in the previous 4 weeks.

Exclusion criteria were: a history of COPD or asthma-COPD overlap (ACO), bronchitis, bronchiectasis, lung cancer, cystic fibrosisor pneumonia; patients with upper airway cough syndrome (UACS), eosinophilic bronchitis (EB) and gastroesophageal reflux-related cough (GERC) [6]; do not continue treatment because of some reasons or diagnosed as other diseases after treatment.

### Ethics statement

The study was conducted in accordance with the Declaration of Helsinki, and the protocol was approved by the Ethics Committee of the Huizhou third people’s Hospital, which absolved the need for written informed consent because of the retrospective study. The ethics protocol number is Nov.46 [2017]. All personal identification data were anonymized and de-identified before analysis.

### Assessments and spirometry

Spirometry, bronchial challenge with methacholine, induced sputum cell differentials and CT of the chest were performed on the same day. Clinical variables were recorded for the patients.

Pre-challenge spirometry: spirometry was performed by Lung Function Machine (MS-pneumo + aps; JAEGER; German). The spirometer measures lung volumes indirectly with a pneumotachograph using the pressure difference by a fine metal mesh. It is sensitive to temperature, humidity and atmospheric pressure of surrounding air and requires constant calibration every day. According to the recommendations of the Chinese National Guidelines of Pulmonary Function Test (2014): the quality and criteria of spirometry, which characteristics of rapid rise in flow/volume curve, duration of expiration more than or equal to 6 s and visualization of peak expiratory flow (PEF) were required. Repeat at least three times (in a reproducible way) and the best was retained. It has been estimated that less than 65% of the small airways must be obstructed before changes can be detected using routine spirometry [16, 17].

BHR test: variable airflow limitation was performed by bronchial challenge with methacholine test and the best spirometric value was measured prior to the methacholine challenge. Patients with a percentage of the predicted forced expiratory volume in first second (FEV_1_%pred) ≥ 60% continued with the challenge (at baseline). The breath dosimeter method was used. The test sequence included five steps: 0.9% NaCl only, 0.078, 0.312, 1.125 and 2.504 mcg. Measure the FEV_1_ at about 60 s from the start of one to the start on the next inhalation from the nebulizer. Obtain an acceptable-quality FEV_1_ at each time point. The procedure was terminated when the FEV_1_ level fell below 20% of the baseline value. The positive response was defined as PD_20_ ≤ 2.504 mcg (between NS and 2.504 mcg). The cumulative dose of PD_20_ was used to assess the degree of BHR [16, 17].

Sputum samples: sputum was induced by single 3% hypertonic saline inhalation through ultrasonic atomizer for 30 min in total. During inhalation, patients were encouraged to stop every 10 min to blow their nose and rinse their mouth, then cough deeply and expectorate sputum into a sterile container. Collected lower respiratory sputum portions were dispersed using 0.1% dithiothreitol with water bath (37℃) and oscillator at 15 min before 300 mesh nylon mesh filter. Total cell counts were centrifuged, smeared and stained (Hematoxylin–Eosin). A differential cell counts were obtained from 400 cells with 400 × microscope to identify the phenotypes of airway inflammation. We defined sputum eosinophilia ≥ 2.5% [6].

### Statistical analysis

All statistical variables were analyzed using SPSS version 22 (IBM Corporation, Armonk, NY, USA). Data were presented as mean ± standard deviation (SD) and percentages. A Student’s *t*-test or Chi-square test was used to observe two groups of CVA and CA patients. Multivariate analysis of variance and analysis of covariance was used to detect the effect on the dependent variable. The relationship between small airways, PD_20_ and sputum cells was detected with the Person correlation coefficient. A *p* value < 0.05 was considered statistically significant.

## Results

A total of 825 asthmatic outpatients attended the diagnosis by pulmonary and critical care medicine physicians based on clinical manifestation, examination findings and a positive response to therapy.

**Fig. 1:**
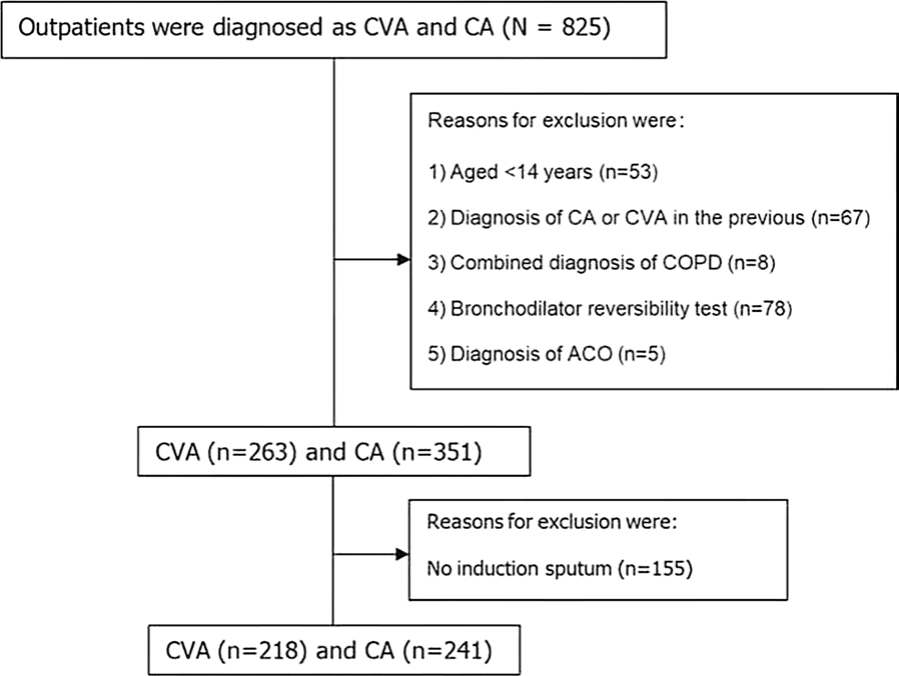
The strategies of flow-chart with asthmatic patients. n: number of subjects

614 asthmatic patients were eligible and analyzed. Reasons for exclusion were (1) aged < 14 years (n = 53); (2) combined diagnosis of COPD or ACO (n = 8 and n = 5, respectively); (3) prior diagnosis of CA or CVA (n = 67); (4) performed by bronchodilator reversibility test (n = 78) or (5) no induction sputum (n = 155). The strategies of the flow chart are illustrated in Fig. 1.

Demographic parameters of the included patients are presented in Table 1. Significant but weaker differences were found in the sex ratio between CVA and CA patients (*p* = 0.041). According to the results of spirometry, FEV_1_%predicted, FEV_1_/ forced expiratory vital capacity (FVC) (FEV_1_/FVC) and PEF%predicted were higher in CVA compared to CA (*p* < 0.05). We did not find any difference in age, BMI and history of smoking between the two groups.

**Table 1:** Baseline characteristics, pre-challenge spirometry and sputum cells of patients with CVA and CA

Demographic parameter	CVA	CA	*p*
n	263	351	–
Age, years	46.06 ± 16.43	48.64 ± 15.85	0.051
Females, n (%)	164, (62.36%)	190, (54.13%)	0.041
BMI, kg/m^2^	23.54 ± 3.62	23.50 ± 3.56	0.902
Smokers, n (%)	56, (21.29%)	95, (27.07%)	0.100
FVC% predicted	97.55 (13.48)	96.47 (12.92)	0.317
FEV_1_ (L)	2.41 (0.65)	2.29 (0.63)	0.023
FEV_1_% predicted	89.21 (11.84)	85.02 (12.36)	< 0.001
FEV_1_/FVC (%)	76.46 (8.62)	73.12 (9.77)	< 0.001
PEF% predicted	89.89 (15.15)	87.10 (16.75)	0.028
MMEF% predicted (< 65%), n (%)	184 (70%)	284 (80.91%)	0.002
FEF_50%_ predicted (< 65%), n (%)	165 (62.74%)	258 (73.5%)	0.004
FEF_75%_ predicted (< 65%), n (%)	199 (75.67%)	277 (78.92%)	0.379
MMEF% predicted	56.10 (18.26)	49.52 (19.57)	< 0.001
FEF_50%_ predicted	60.54 (19.46)	52.76 (20.16)	< 0.001
FEF_75%_ predicted	53.37 (20.18)	47.10 (22.92)	< 0.001
PD_20_ (mcg)	0.89 (0.79)	0.71 (0.8)	0.005
FEV_1_ fall (%)	26.95 (7.92)	29.65 (11.48)	0.001
n	241	218	–
Sputum eosinophils, %	9.32 ± 14.37	13.57 ± 19.76	0.009
Sputum neutrophils, %	74.56 ± 20.45	69.25 ± 22.89	0.008

N refers to the total patients; n refers to the sub-group population; Data are presented as mean ± SD (standard deviation) and frequencies

CVA, cough variant asthma; CA, classic asthma; BMI, body mass index; FVC, forced vital capacity; FEV_1_, forced expiratory volume in 1 second; PEF, peak expiratory flow; MMEF, maximum mid-expiratory flow; FEF, forced expiratory flow; PD_20_ , fall of FEV_1_ by 20% of pre-challenge value; the difference between groups was analyzed by Student’s t-test or Chi square. Significant *p* value < 0.05

Patients with CVA showed less small airway dysfunction (< 65%) than the CA group in MMEF%predicted (70% vs 80.91%, *p* = 0.002) and FEF_50%_predicted (62.71% vs 73.5%, *p* = 0.004). The function of small airways was significantly higher in the CVA group compared with the CA group (*p* < 0.001) (Table 1).

Response to BHR in CVA and CA are showed in Table 1. Significantly higher of BHR was found in CA patients than those in CVA patients (*p* = 0.005). Compared to CVA, CA showed more sensitivity in the degree of FEV_1_ fall (%) (*p* = 0.002 and *p* = 0.001, respectively).

Sputum eosinophilia was defined as ≥ 2.5%according the recommendations of China [6]. CVA or CA patients were divided into the eosinophilic airway inflammatory subtype group and the non- eosinophilic airway inflammatory subtype group. In the eosinophilic subtype of CVA or CA, patients had lower PD_20_ and proportion of neutrophils compare to the diseases of non-eosinophilic subtype (*p* ≤ 0.01). Small airways (MMEF%predicted, FEF_50%_predicted and FEF_75%_predicted) were higher in the non-eosinophilic subtype of CA (*p* < 0.05), Proportion of sputum eosinophils were less in CVA both in eosinophilic airway inflammation of CVA and CV and in non-eosinophilic airway inflammation of CVA and CA (Table 2).

**Table 2:** Small airways, BHR and induction sputum in CVA and CA

	CA-Sputum eosinophilia	CA-Sputum non-eosinophilia	CVA-Sputum eosinophilia	CVA-Sputum non-eosinophilia	*p* values			
					CA-SE vs CA-NSE	CVA-SE vs CVA-NSE	CA-SE vs CVA-SE	CA-SNE vs CVA-SNE
n	132	86	126	115				
MMEF% predicted	45.75 ± 17.49	51.14 ± 19.00	54.11 ± 19.83	55 ± 17.22	0.036	0.71	< 0.001	0.133
FEF_50%_ predicted	49.94 ± 18.6	55.46 ± 19.7	58.08 ± 20.12	58.91 ± 19.52	0.038	0.748	0.001	0.22
FEF_75%_ predicted	40.95 ± 19.51	47.58 ± 21.31	50.92 ± 21.73	50.25 ± 19.60	0.019	0.803	< 0.001	0.359
PD_20_ (mcg)	0.46 ± 0.66	0.90 ± 0.83	0.60 ± 0.72	0.93 ± 0.82	< 0.001	0.001	0.006	0.802
Sputum eosinophils, %^a^	21.81 ± 21.72	0.77 ± 0.77	17.14 ± 16.26	0.91 ± 0.72	–	–	0.048	0.032
Sputum neutrophils, %^a^	61.68 ± 23.16	81.00 ± 16.33	67.44 ± 21.54	82.54 ± 14.89	< 0.001	< 0.001	0.032	0.442

MMEF, maximum mid-expiratory flow; FEF, forced expiratory flow; PD_20_, fall of FEV_1_ by 20% of pre-challenge value; CA-SE, CA-Sputum eosinophilia; CA-SNE, CA-Sputum non-eosinophilia; CVA-SE, CVA-Sputum eosinophilia; CVA-SNE, CVA-Sputum non-eosinophilia. Data are expressed as the mean ± SD; the difference between groups was analyzed by Student’s t-test or analysis of covariance. *p* < 0.05 versus the control; ^a^Analysis of covariance, ages as a covariance variable.

**Fig. 2:**
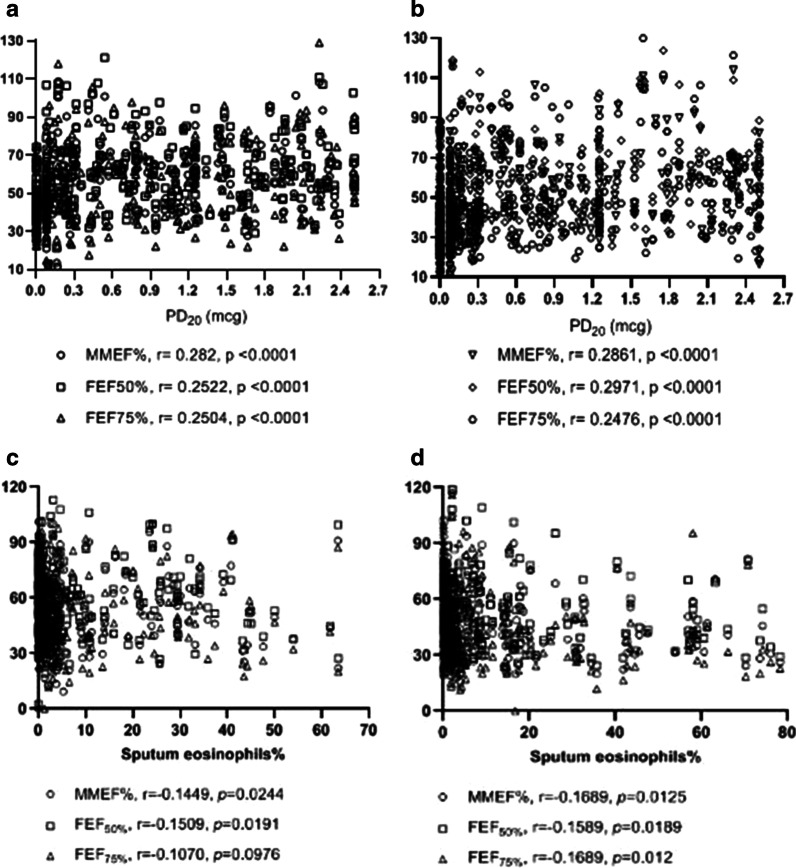
Scatter plots of correlation between small airways and PD_20_, the percentage of sputum eosinophils. **a** Correlation between PD_20_ (mcg) and small airways (MMEF%, FEF_50%_, FEF_75%_) in CVA; **b** Correlation between PD_20_ (mcg) and small airways (MMEF%, FEF_50%_, FEF_75%_) in CA; **c** Correlation between the percentage of sputum eosinophils and small airways (MMEF%, FEF_50%_, FEF_75%_) in CVA; **d** Correlation between the percentage of sputum eosinophils and small airways (MMEF%, FEF_50%_, FEF_75%_) in CA

Figure 2 gives an overview of all correlations between small air ways (MMEF%, FEF_50%_, FEF_75%_) and PD_20_, sputum eosinophils% with CVA and CA. Significant positive correlations were observed for PD_20_ and MMEF% predicted (r = 0.282, *p* < 0.001), FEF_50%_predicted (r = 0.2522, *p* < 0.001), FEF_75%_predicted (r = 0.2504, *p* < 0.001) in patients with CVA (Fig. 2a). We also found significant correlations between sputum eosinophils% and MMEF% predicted (r = − 0.1449, *p* = 0.0244), FEF_50%_predicted (r = − 0.1509, *p* = 0.0191) with CVA patients (*p* < 0.05) (Fig. 2c). Significant association between small airways and PD_20_, sputum eosinophils% was also analyzed in CA (*p* < 0.05) (Fig. 2b, d).

**Fig. 3:**
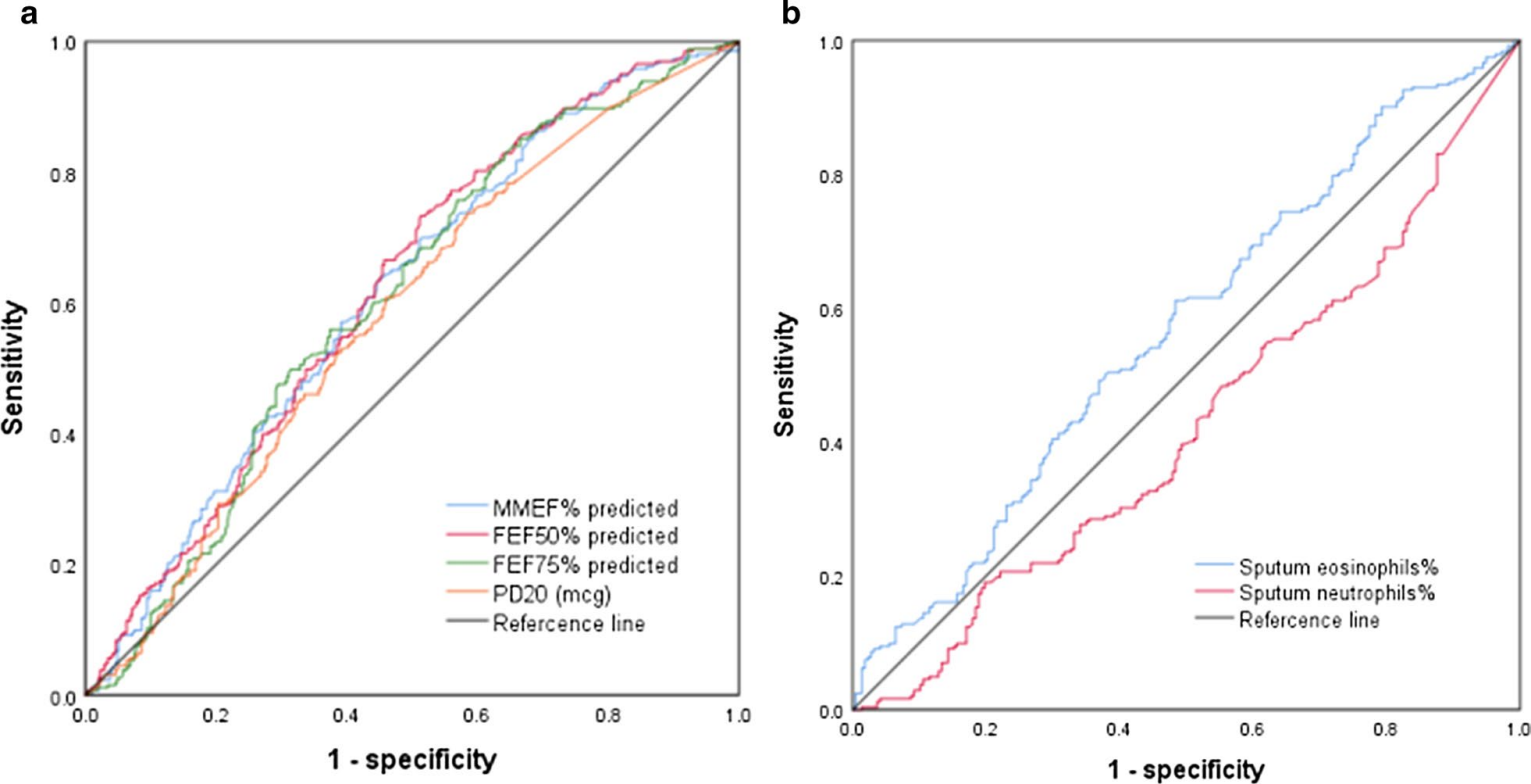
ROC curve analysis for CVA versus CA. **a** PD_20_, MMEF, FEF_50_, FEF_75_ (%predicted) or CVA diagnosis; **b** the percentage of sputum cells for CVA diagnosis

**Fig. 4:**
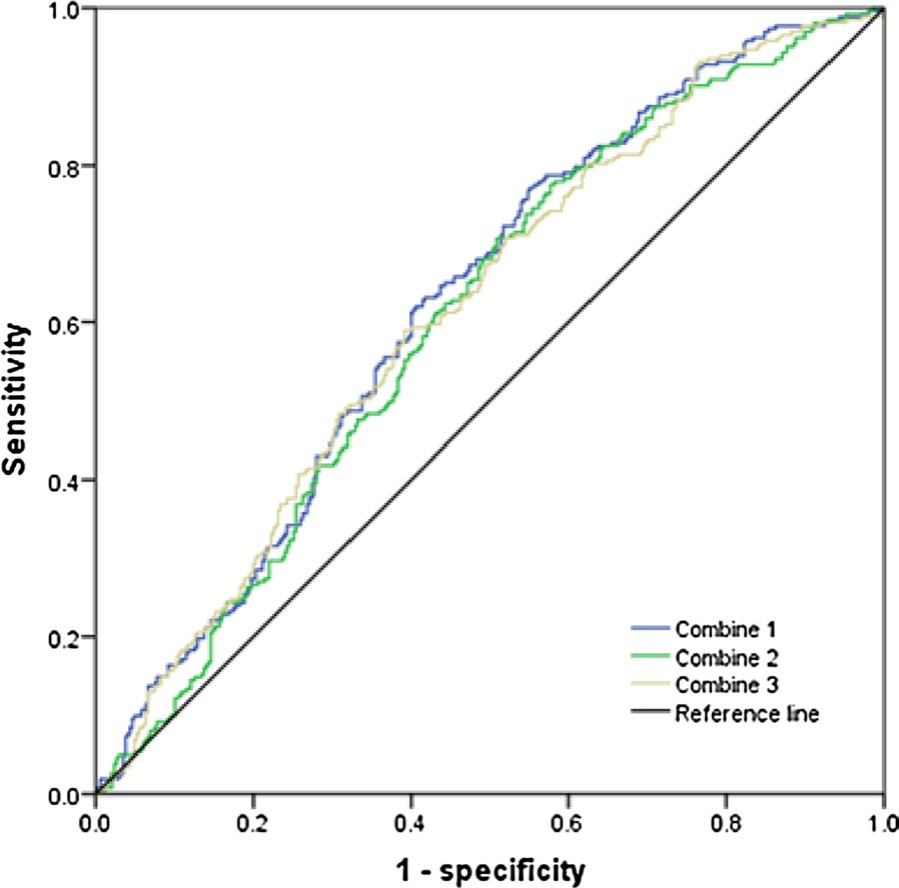
Predictive value for CVA by combining PD_20_ and small airway resistance; combination 1: PD_20_ + FEF_50_ (% predicted); combination 2: PD_20_ + FEF_75_ (% predicted); combination 3: PD_20_ + MMEF (% predicted)

The ROC curve of Table 3 and Fig. 3 presented small airways (MMEF%, FEF_50%_, FEF_75%_), PD_20_ and sputum eosinophils% as predictors to identified CVA from CA. The optimum cut-point for sputum eosinophils% was 4.7% with an area under the curve (AUC) of 0.575 (*p* = 0.005), and the AUC of MMEF, FEF_50_ and FEF_75_ (%predicted) was 0.615, 0.621, 0.606, respectively. In addition, 0.17mcg of PD_20_ was the best diagnostic value for CVA with an AUC of 0.582 (*p* = 0.001). Data of sensitivity and specificity were showed in Table 3. The AUC of PD_20_ combined with MMEF% predicted was 0.616, and that combined with MEF_50%_predicted and MEF_75%_predicted was 0.625, 0.606, respectively (Fig. 4).

**Table 3:** Results of ROC analysis of PD_20_ and spirometry

Parameter	PD_20_ (mcg)	Sputum eosinophils%	MMEF% predicted	FEF_50%_ predicted	FEF_75%_ predicted
CVA versus CA					
AUC (95% CI)	0.582	0.575	0.615	0.621	0.606
Cut-off	0.17	4.7	47.85%	47.95%	38.2%
Sensitivity/specificity (%)	73.8/41.4	65.7/52.1	63.9/54.9	73.4/48.6	75.7/43.1
*p*-value	0.001	0.005	< 0.001	< 0.001	< 0.001
95% confidence interval					
Upper limit value	0.627	0.628	0.659	0.665	0.649
Lower limit value	0.537	0.523	0.570	0.577	0.560

CVA, cough variant asthma; CA, classic asthma; PD_20_, Does of methacholine induce a 20% decrease in FEV_1_. Significant *p* value < 0.05.

## Discussion

The retrospective study showed that CVA patients were different with in proportion of sex, smoker, spirometry, BHR and proportion of sputum cells, compared to CA patients. Lower sputum eosinophilia, milder BHR and small airway dysfunction were showed in CVA, and the correlation between them was observed weak but significant differences. The eosinophilic airway inflammation of CA showed severer small airway dysfunction compared to the non-eosinophilic airway inflammation of CA and the eosinophilic airway inflammation of CVA, respectively. Small airways, PD_20_ and sputum eosinophils were poor to distinguish patients with CVA. We had also evaluated the value of PD_20_ combined with small airways in CVA prediction, which reflected significant, but not enough to be clinically useful.

Chronic cough is the sole presenting symptom of CVA. The chronic persistent non-productive cough is more common in females, and females are more easily troubled by the symptom [18, 19]. The cough threshold is lower in females than in males, illustrating that the cough sensitivity is heightened in females [20, 21]. In our study, the difference in gender is weak and the number of female was a little frequent in CVA (*p* = 0.041). Additional studies are needed to better understand gender differences.

Spirometry is the fundamental diagnostic method and is easy to assess the airflow limitation associated with asthma. Parameters such as FEV_1_ and PEF are frequently used to evaluate proximal airway obstruction. In the study, we found that patients with CVA have a better FEV_1_predicted and PEF%predicted (*p* < 0.05). Spirometric values indices are almost independent of the patient’s activity if the expiration is forced; they depend only on the properties of the respiratory system because of the airflow limitation phenomenon [22–24]. Evaluation of forced spirometry results begins with an analysis of whether bronchial airflow capacity is quantified through the FEV_1_.

Previously, asthma was understood to be a disease primarily of the central airways. However, surgical lung specimens with living chronic asthma and autopsy specimens with fatal asthma reveal mucus plugging and inflammatory involvement of both the small and large airways [25, 26]. An inflammatory characterized by increased T cells, activated eosinophils and major basic protein in the small airways, which was similar to the inflammation of the central airways [26]. The intensity of the inflammation may be even higher in the small airways compared with central airways [27]. These observation confirm that the chronic inflammation of asthma involves the entire lung, from the large proximal to the small distal airways. Flow measures of small airways commonly used in forced expiratory flow at 50% (FEF_50%_), 75% (FEF_75%_) and 25–75% (FEF_25–75%_/MMEF) of FVC. Among these parameters, FEF_25–75%_ is the most commonly adopted, although the literature supporting its reliability is not conclusive.

The pathophysiological features of CVA are similar to those of CA. CVA shows similar levels of eosinophilic airway inflammation and a milder degree of airway remodeling, such as subepithelial thickening, goblet cell hyperplasia, and vascular proliferation [28–30]. Nearly 30% of CVA patients eventually develop to CA, sometimes severe enough to require continuous treatment [5, 31]. Given these studies, CVA is considered to be the initial stage of asthma [32]. Therefore, early diagnosis and treatment are recommended to attenuate the inflammation and remodeling. In our study, the spirometric parameters for small airways with the CVA group were better than those with the CA group. Small airway dysfunction was present in a large proportion of asthma patients at baseline, and less in CVA compared to those of CA. The eosinophilic airway inflammation of CA showed severer small airway dysfunction. A positive relationship between small airways and PD_20_, induced sputum cells in both CVA and CA. The optimum cut-point for MMEF, FEF_50%_predicted and FEF_75%_predicted were 47.85%, 47.79% and 38.2%, and the AUC of them were 0.615, 0.621 and 0.606, respectively. 0.17 mcg of PD_20_ and 4.7% of sputum eosinophilia may help identify CVA with an AUC of 0.582 and 0.575 (*p* = 0.001 and *p* = 0.005, respectively). The correlation and ROC analyses demonstrated a relatively poor, although significant relationship for small airways, PD_20_ and induced sputum cells to predict CVA in statistics.

## Limitation of the study

A positive relationship showed between small airways and PD_20_, sputum cells. However, overall the association appeared weak and low AUCs for the prediction to CVA. While these correlations might show statistical significance, none of these appear convincing and potentially clinically relevant judged. Similarly, the low AUC values are highly unreliable and not helpful to inform clinical decision making.

## Conclusions

This study showed that lower sputum eosinophilia, milder small airway obstruction and BHR are in CVA. The eosinophilic airway inflammatory subtype of CA showed severer small airway dysfunction. However, the relationship between them is poor. Small airways and BHR, induction sputum cells may not be used to detect CVA patients. Based on these weak correlations and prediction values, further investigations would be required.

## Data Availability

Due to the institutional policy, the datasets used and/or analysed during the current study are available from the corresponding author on reasonable request.

## Abbreviations

BHR: Bronchial hyper-responsiveness
GINA: The Global Initiative for Asthma
FVC: Forced vital capacity
FEV_1_: Forced expiratory volume in 1 s
PEF: Peak expiratory flow
MMEF: Maximum mid expiratory flow
MEF: Maximal expiratory flow
CVA: Cough variant asthma
CA: Classic asthma
